# In vitro genotoxicity studies: n-Butyl acrylate L5178Y mouse lymphoma (TK+/− locus assay), 2-Ethylhexyl acrylate gene mutation assay in Chinese hamster V79 cells, and 2-Ethylhexyl acrylate micronucleus test in human lymphocytes

**DOI:** 10.1016/j.dib.2018.06.008

**Published:** 2018-06-14

**Authors:** Sandra Murphy, Robert Ellis-Hutchings, Lavorgie Finch, Stefanie Welz, Karin Wiench

**Affiliations:** a Kennett Square, PA 19348, United States; b The Dow Chemical Company, Midland, MI 48674, United States; c Arkema Inc., King of Prussia, PA 19406, United States; d BASF SE, Ludwigshafen am Rhein, Germany

## Abstract

Available point mutation tests have shown inconsistent results with various acrylates. Most of those tests were performed prior to OECD guidelines and appropriate data regarding cytotoxicity are not given. Data from three current OECD guideline compliant experiments conducted under GLP are provided. They include (a) an in vitro mouse lymphoma (TK+/−) assay (OECD 490) [3], (b) an in vitro HPRT locus gene mutation assay utilizing cultures of Chinese hamster V79 cells (OECD 476) [1], and (c) an in vitro micronucleus test in human lymphocytes (OECD 487) [2]. Test materials were not mutagenic under these experimental conditions, adding to the weight-of-evidence of non-genotoxicity for this group of chemicals.

**Specifications Table**

**Table:** 

Subject area	Toxicology
More specific subject area	in vitro genotoxicity
Type of data	Data tables and methods summaries
How data was acquired	Laboratory experiments using current health effects guidelines.
Data format	Derived from the final laboratory reports.
Experimental factors	See method below
Experimental features	Studies performed under GLP conditions according the current OECD Test guideline 490 “In Vitro Mammalian Cell Gene Mutation Tests Using the Thymidine Kinase Gene” (Mouse Lymphoma Assay), OECD Test Guideline 476 “In Vitro Mammalian Cell Gene Mutation Tests Using The Hprt and Xprt Genes”(HPRT Test), and OECD Test Guideline 487 “In Vitro Mammalian Cell Micronucleus Test” Using Human Lymphocytes
Data source location	Mouse Lymphoma Assay was conducted at the Experimental Toxicology and Ecology Laboratories of BASF SE, Ludwigshafen, Germany; the HPRT Assay in V79 Cells and in vitro Micronucleus Test were conducted at Envigo CRS GmbH, Rossdorf, Germany
Data accessibility	Data are provided below.
Related research article	Impact of strain and maximum tolerated dose (MTD) selection in dermal carcinogenicity studies conducted for hazard assessment of non-genotoxic irritants. 2-Ethylhexyl acrylate as a case study.

**Value of the data**

• Genotoxicity is an important determinant in the mode of action of a chemical and important in human hazard assessments, such as that recently conducted on 2-Ethylhexyl acrylate-induced skin tumorigenesis [1].
• Older genotoxicity tests showed inconsistent results with various acrylates. Most of those tests were performed prior to OECD guidelines and appropriate data regarding cytotoxicity are not given.
• Three new in vitro genotoxicity studies conducted according to current OECD guidelines (i.e., mouse lymphoma-TG 490 [2], HPRT-TG 476 [3], and micronucleus-TG 487 [4]) did not show genotoxic activity under these experimental conditions, adding to the weight-of-evidence of non-genotoxicity for this group of chemicals.

## 1 Data, experimental design, materials, and methods

### 1.1 n-Butyl acrylate mouse lymphoma assay

An in vitro gene mutation test in L5178Y mouse lymphoma cells was conducted under GLP according to OECD Guideline 490 [2], to evaluate the ability to induce gene mutations at the thymidine kinase (TK) locus or structural chromosome aberrations at chromosome 11 in L5178Y TK+/− mouse lymphoma cells with the microwell method.

*Test substance*: n-butyl acrylate (nBA) (CAS # 141-32-2) 99.7%.

*Positive controls*: Methyl methansulfonate (MMS) (CAS # 66-27-3) 15 μg/mL (4-h exposure -S9) and 5 μg/mL (24-h exposure, -S9); cyclophosphamide (CPP) (CAS# 50-18-0) 2.5 μg/mL; 7,12-dimethylbenz[a]anthracene (DMBA) (CAS # 57–97-6) 1.0 and 2.5 μg/mL.

*Vehicle*: Dimethylsulfoxide (DMSO) (CAS # 67-68-5) for nBA and DMBA.

*Metabolic activation*: Phenobarbital and β-naphthoflavone induced rat S9 fraction was prepared at the laboratory and frozen until needed; fresh S9 mix was prepared prior to each experiment.

*Cell culture*: L5178Y TK+/− mouse lymphoma cells were removed from frozen stock; thawed and incubated in medium for a day, resuspended in fresh medium and incubated until used.

*Treatment*: Cells were treated with the test substance for 4 and 24 h without S9 and 4 h with S9. Cells were then cultured for an expression period of about 48 h and then cultured in selection medium for approx. 10 days, after which the number of large and small colonies was determined.

In this study all incubations were performed at 37 °C with a relative humidity of ≥ 90% in a 5% (v/v) CO_2_ atmosphere.

*Scoring and data analysis*: The number of empty wells of the 96-well plates was scored and recorded. To account for any loss of cells during the 4-h treatment, a relative growth during treatment factor (RGDT, %) was calculated by comparing the growth of each treated culture relative to the control. The colonies were classified into large colonies (indication of gene mutation) and small colonies (indication of chromosome breakage). An appropriate statistical method to test for linear trend was performed to assess a possible linear dose-relation in mutant frequencies and judged statistically significant whenever the one-sided *p*-value (probability value) was < 0.05 and the slope > 0. Both biological and statistical significance have been considered together.

The cloning efficiency (CE, %) was calculated for each test group as follows:

$$\mathrm{CEx}=\frac{-\;\ln\;[\mathrm{total}\;\mathrm{number}\;\mathrm{of}\;\mathrm{empty}\;\mathrm{wells}/\mathrm{total}\;\mathrm{number}\;\mathrm{of}\;\mathrm{seeded}\;\mathrm{wells}]}{\mathrm{number}\;\mathrm{of}\;\mathrm{seeded}\;\mathrm{cells}\;\mathrm{per}\;\mathrm{well}}\times 100$$

RCEx=[CExofthetestgroup/CExofthevehiclecontrol]×100

Mutant frequency was calculated

$${\mathrm{MF}}_{\mathrm{Uncorrected}}=\frac{-\;\ln\;[\mathrm{total}\;\mathrm{number}\;\mathrm{of}\;\mathrm{empty}\;\mathrm{wells}/\mathrm{total}\;\mathrm{number}\;\mathrm{of}\;\mathrm{seeded}\;\mathrm{wells}]}{\mathrm{Number}\;\mathrm{of}\;\mathrm{seeded}\;\mathrm{cells}\;\mathrm{per}\;\mathrm{well}}\times 10^{6}$$

$${\mathrm{MF}}_{\mathrm{corrected}}={\mathrm{MF}}_{\mathrm{uncorrected}}/\mathrm{CE}2\times 100$$

Positive and negative controls reacted as expected supporting test validity. Cytotoxicity indicated by reduced relative total growth (RTG) of below 20% of control was observed in all experiments in the absence and presence of metabolic activation, except in the first experiment with metabolic activation. A third experiment was added to comply with current guidelines. nBA did not cause any biologically relevant increase in the mutant frequencies either without S9 mix or after adding a metabolizing system in three experiments performed independently of each other. Linear dose response relationships determined to be statistically significant were biologically irrelevant if the corrected mutant frequencies did not exceed the corresponding global evaluation factor. (Table 1, Table 2, Table 3).

**Table 1:** n-Butyl acrylate cytotoxicity and mutant frequency without S9.

**Duration**	**Dose**	**Cytotoxicity**^a^		**Genotoxicity**	
**h**	**µg/mL**	**RCE1%**	**RTG %**	**MFc**	**MFT**
4	1% DMSO^b^	100.0	100.0	52.7	179
	2.34	98.5	84.0	49.5	
	4.69	80.6	50.8	49.8	
	9.38	95.5	88.8	40.8	
	18.75	72.4	26.0	69.8	
	37.50	2.5	n.c.	n.c.	
	75.00	1.4	n.c.	n.c.	
	MMS 15 µg/ml^c^	77.2	34.3	1074.7	
24	1% DMSO^b^	100	100	75.7	202
	1.56	100.9	91.3	53.1	
	3.13	95	68.4	48.0	
	6.25	94.2	81.1	26.4	
	12.5	63.5	37.3	62.2	
	25	12.5	n.c.	n.c.	
	50	1.5	n.c.	n.c.	
	MMS 5 µg/ml^c^	78	46.2	572.7	

MFc - Corrected mutant frequency: mutant colonies per 10^6^ cells corrected with the CE2 (viability) value.

MFT - Mutant Frequency Threshold (GEF): number of mutant colonies per 10^6^ cells of current vehicle control plus 126 (rounded value).

n.c. Culture was not continued due to strong cytotoxicity.

a Cytotoxicity related to the respective vehicle control: RCE - relative cloning efficiency (survival); RTG - Relative total growth.

b Vehicle control.

c Positive control.

**Table 2:** n-Butyl acrylate cytotoxicity and mutant frequency with S9 (4 h).

**Experiment**	**Dose**	**Cytotoxicity**^a^		**Genotoxicity**	
	**μg/mL**	**RCE1%**	**RTG %**	**MFc**	**MFT**
I	1% DMSO^b^	100.0	100.0	45.4	171
	9.38	96.4	70.2	55.2	
	18.75	87.7	68.5	59.4	
	37.50	118.6	72.1	40.1	
	75.00	96.4	54.8	43.2	
	150.00	103.8	53.0	35.4	
	300.00	65.0	36.3	47.6	
	CPP 2.5 µg/ml^c^	81.6	35.6	777.4	
	DMBA 1.0 µg/ml^c^	66.4	20.4	955.2	
	DMBA 2.5 µg/ml^c^	84.0	38.6	698.3	
II	1% DMSO^b^	100.0	100.0	37.2	163
	25.00	102.2	91.5	33.5	
	50.00	117.3	79.9	35.4	
	100.00	107.7	60.2	28.6	
	200.00	102.2	67.4	31.0	
	400.00	45.1	19.4	101.0	
	800.00	2.6	n.c.	n.c.	
	CPP 2.5	73.5	53.2	228.3	
	DMBA 1.0 µg/ml^c^	52.7	59.7	261.9	
	DMBA 2.5 µg/ml^c^	58.4	38.9	362.5	
III	1% DMSO^b^	100.0	100.0	48.6	175
	18.75	98.5	76.0	48.2	
	37.50	90.6	68.8	44.2	
	75.00	91.9	54.0	43.8	
	150.00	78.2	58.1	44.9	
	300.00	47.7	20.3	91.5	
	600.00	1.9	n.c.	n.c.	
	900.00	0.7	n.c.	n.c.	
	CPP 2.5 µg/ml^c^	66.9	47.9	362.4	
	DMBA 1.0 µg/ml^c^	73.9	42.2	356.2	
	DMBA 2.5 µg/ml^c^	82.3	49.4	299.3	

MFc - Corrected mutant Frequency: mutant colonies per 106 cells corrected with the CE2 value.

MFT - Mutant Frequency Threshold (GEF): number of mutant colonies per 106 cells of current vehicle control plus 126 (rounded value).

n.c. Culture was not continued due to strong cytotoxicity.

a Cytotoxicity related to the respective vehicle control: RCE - relative cloning efficiency; RTG - Relative total growth.

b Vehicle control.

c Positive control.

**Table 3:** Statistical analysis – linear trend test.

Corrected mutation frequency	Slope	One sided *p* value*
1st Experiment without S9 mix	**2.03954**	**0.0379**
1st Experiment with S9 mix	− 0.30324	0.6165
2nd Experiment without S9 mix	− 0.4728	0.6755
2nd Experiment with S9 mix	**4.60547**	**0.0005**
3rd Experiment with S9 mix	**2.58994**	**0.0135**

Values indicating a statistically significant trend are printed in bold characters.

* The linear trend-test testing for an increased mutant frequency is significant (significance level of 5%), if the one-sided *p*-value is lower than 0.05 and the slope is greater than 0.

## 1.2 2-Ethylhexyl acrylate HPRT assay in Chinese hamster V79 cells

An in vitro genotoxicity experiment was conducted using 2EHA, under GLP and in accordance with OECD Guideline 476 [3]: The in vitro assay to investigate the potential to induce gene mutations at the HPRT locus in cultures of Chinese hamster V79 cells.

*Test substance*: 2-ethylhexyl acrylate (2EHA) (CAS# 103-11-7) 99.7%.

*Vehicle*: Ethanol (EtOH), purity 99.9% (CAS# 64-17-5).

*Positive controls*: Ethylmethane sulfonate 99% (EMS) (CAS# 62-50-0) in nutrient medium; 7,12-dimethylbenz(a)anthracene ≥ 95% (DMBA) (CAS# 57-97-6) in DMSO (CAS # 67-68-5) vehicle (final concentration in nutrient medium 0.5%).

*Metabolic activation*: phenobarbital/β-naphthoflavone induced rat liver S9 supernatant mixed with S9 cofactor solution for a final protein concentration of 0.75 mg/mL in the cultures.

*Cell cultures*: The V79 cell line was obtained from the stored cell bank of the test laboratory. For seeding of the cell cultures, the complete culture medium was minimal essential medium containing Hanks salts, neomycin (5 µg/mL), 10% fetal bovine serum (FBS), and amphotericin B (1%). During treatment no FBS was added to the medium. For the selection of mutant cells the complete medium was supplemented with 11 µg/mL 6-thioguanine. All incubations were done at 37 °C with 1.5% carbon dioxide (CO_2_) in humidified air.

Doses were selected based on pretests in the presence and absence (4 h treatment) of metabolic activation.

*Scoring and data analysis*: A linear regression was performed to assess a possible dose dependent increase of mutant frequencies. The numbers of mutant colonies generated with the test item were compared to the solvent control groups. A trend is judged as significant whenever the *p*-value (probability value) is below 0.05.

*Treatment*: Test item concentrations between 14.4 µg/mL and 1843.0 µg/mL (~ 10 mM) were used. Due to phase separation the following test concentrations were selected: without metabolic activation: 14.4, 28.8, 57.6, and 115.2 µg/mL; with metabolic activation: 14.4, 28.8, 57.6, 115.2, and 230.4 µg/mL. No relevant cytotoxic effect occurred up to the highest concentration with and without metabolic activation. A single cell suspension was prepared from phosphate buffered saline (PBS) rinsed cells, then trypsinized in complete culture medium with 10% FBS. The cells were grown for 24 h prior to treatment. After 24 h the medium was replaced with serum-free medium containing the test item, with or without S9. Concurrent solvent and positive controls were treated in parallel. 4 h after treatment, this medium was replaced with complete medium following two washing steps with PBS. Immediately after treatment the cells were trypsinized and sub-cultivated, and then again in 3 days before final seeding of cultures that were stained and evaluated. Cloning efficiency was evaluated after each subcultivation using additional flasks seeded to determine the relative survival (RS) as measure of test item induced cytotoxicity. The cultures were incubated at 37 °C in a humidified atmosphere with 1.5% CO2 for about 8 days. The colonies were stained with 10% methylene blue in 0.01% KOH solution. The stained colonies with more than 50 cells were counted.

No substantial and reproducible dose dependent increase of the mutation frequency was observed in the main experiment. Positive controls induced a distinct increase in mutant colonies and, thus, showed the sensitivity of the test system and the activity of the metabolic activation system Table 4, Table 5, Table 6.

**Table 4:** Toxicity Data 2EHA V79 HPRT Assay Pre-test (4hr).

**Test substance**	**Conc. (ug/mL)**	**Cloning Efficiency absolute (%)**	**Cloning Efficiency relative (%)**	**P/Ps**
EtOH	0.50%	71	100	-
2EHA	14.4	76.7	108.1	-
	28.8	71.5	100.8	-
	57.6	68.7	96.7	-
	115.2	71.9	101.3	-
	230.4	67.8	95.6	-
	460.8	65.9	92.8	-
	921.5	62.6	88.2	-
	1843	64.8	91.4	-
EtOH +S9	0.50%	93	100	-
2EHA +S9	14.4	79	85	-
	28.8	72.2	77.6	-
	57.6	72.2	77.6	-
	115.2	79.8	85.9	-
	230.4	84.6	91	-
	460.8	69.4	74.6	-
	921.5	72	77.4	-
	1843	71.7	77.1	-

P/PS - Precipitation / Phase separation

**Table 5:** Cloning Efficiency – 2EHA V79 HPRT Assay Main Experiment.

**Test sub-stance**	**Conc. μg/ mL**	**PS**	**Culture I Cloning Efficiency**					**Culture II Cloning Efficiency**				
			**1 - survival**			**2- viability**		**1 - survival**			**2- viability**	
			**Abs**	**Rel (%)**	**Cell density (%)**	**Abs**	**Rel (%)**	**Abs**	**Rel (%)**	**Cell density (%)**	**Abs**	**Rel (%)**
EtOH	0.5%	-	0.6	100	100	0.7	100	0.6	100	100	0.5	100
2EHA -S9	14.4	-	0.5	85.3	97.5	0.6	89.5	0.5	75	77.8	0.5	111
	28.8	-	0.5	79.8	95.1	0.6	83.9	0.6	86.3	91.1	0.5	94.7
	57.6	-	0.4	73	83.8	0.6	92.2	0.5	76.1	74.3	0.4	89
	115.2^a^	+	0.4	65.2	78.9	0.7	98.9	0.4	69.8	85.1	0.5	106.2
EMS	300	-	0.5	81.8	106.3	0.6	85	0.5	84.3	77.1	0.4	84.5
EtOH +S9	0.5%	-	0.6	100	100	0.5	100	0.6	100	100	0.5	100
2EHA +S9	14.4	-	0.7	112.2	72.8	0.5	92.2	0.6	98.1	103.2	0.5	87
	28.8	-	0.6	107	73.5	0.4	72.2	0.6	101.2	100.1	0.5	85.8
	57.6	-	0.5	88.2	68	0.6	109.5	0.7	111.5	100.1	0.5	98.5
	115.2	-	0.6	102.6	63.6	0.4	84.6	0.6	103.6	97.8	0.5	88
	230.4^b^	+	0.5	80.3	80.8	0.5	97.9	0.6	99.6	100.4	0.7	122.5
DMBA +S9	2.3	-	0.5	91.4	77.8	0.4	83.5	0.6	102.3	94.5	0.5	89.6

PS – Phase Separation

a cultures @ 230.4, 460.8, 921.5, 1843 not continued to avoid analysis of too many phase separating concentrations

b cultures @ 460.8, 921.5, 1843 not continued to avoid analysis of too many phase separating concentrations

**Table 6:** 2-Ethylhexyl Acrylate Summary of Experimental Mutation Data V79 HPRT Assay.

Treatment	Concentration (µg/mL)	Rel adjusted cloning efficiency	Mutant colonies/ 10^6^ cells	95% confidence interval
EtOH	0.50%	100	21.7	1.7 – 30.2
2EHA	14.4	70.8	28.8	1.7 – 30.2
	28.8	77.3	24.9	1.7 – 30.2
	57.6	58.9	21.1	1.7 – 30.2
	115.2	55.4	20.6	1.7 – 30.2
EMS	300	76	327.8	1.7 – 30.2
EtOH +S9	0.5%	100	23.1	2.0 – 29.4
2EHA +S9	14.4	91.5	25.9	2.0 – 29.4
	28.8	90.0	25.4	2.0 – 29.4
	57.6	85.8	24	2.0 – 29.4
	115.2	83.3	21.7	2.0 – 29.4
	230.4	82.5	22.5	2.0 – 29.4
DMBA +S9	2.3	83.9	213.5	2.0 – 29.4

## 1.3 2-Ethylhexyl acrylate in vitro mammalian cell micronucleus test in human lymphocytes

An in vitro genotoxicity experiment was conducted using 2EHA, under GLP and in accordance with OECD Guideline 487 [4]: in vitro mammalian cell micronucleus test assay to investigate the potential to induce micronuclei associated with chromosome damage in cultures of human lymphocyte cells.

*Test substance*: 2-ethylhexyl acrylate (2EHA) (CAS# 103-11-7) 99.7%.

*Vehicle*: Acetone, purity 99.98% (CAS# 67-64-1).

*Positive controls*: Mitomycin C (MMC) 98% (CAS# 50-07-7) 0.8 µg/mL aqueous; demecolcine ≥ 98% (CAS # 477-30-5) 100 ng/mL aqueous; with metabolic activation: cyclophosphamide 97.0–103.0% (CPA) (CAS# 50-18-0) 17.5 µg/mL in aqueous saline (0.9% NaCl).

*Metabolic activation*: Phenobarbital/β-naphthoflavone induced rat liver S9 supernatant mixed with S9 cofactor solution for a final protein concentration of 0.75 mg/mL in the cultures.

*Cell culture*: Blood was obtained from healthy non-smoking donors not receiving medication, ages between 27–25 yrs, who had previously established low incidence of micronuclei in their peripheral blood lymphocytes. Blood culture: An 11% mixture of whole blood in Dulbecco׳s Modified Eagles Medium/Ham׳s F12 (DMEM/F12, mixture 1:1) with 200 mM GlutaMAX, penicillin/streptomycin (100 U/mL/100 µg/mL), the mitogen PHA (3 µg/mL), 10% FBS (fetal bovine serum), 10 mM HEPES and the heparin (125 U.S.P.-U/mL).

*Treatment*: All incubations were done at 37 °C with 5.5% CO_2_ in humidified air. Preparation time for all trials was 40 h: 4 h+16 h recovery, or 20 h continuous exposure, followed by 20 h of Cytochalasin B (CAS# 14930-96-2) exposure.

*Scoring and data analysis*: At least 1000 binucleate cells per culture were scored for cytogenetic damage on coded slides. The frequency of micronucleated cells was reported as % micronucleated cells. To describe a cytotoxic effect the CBPI was determined in 500 cells per culture and cytotoxicity is expressed as % cytostasis. A CBPI of 1 (all cells are mononucleate) is equivalent to 100% cytostasis. Statistical significance was confirmed by the Chi square test (*α* < 0.05) for those values that indicated an increase in the number of cells with micronuclei compared to the concurrent solvent control. A linear regression assessed possible dose dependency in the rates of micronucleated cells in test groups compared to the solvent controls. A trend is judged as significant whenever the *p*-value (probability value) is below 0.05. Biological and statistical significance were considered together.

*Interpretation of results*: Test substance is not clastogenic and non-aneugenic if: (a) no concentration exhibits a statistically significant increase compared with the concurrent solvent control; (b) there is no concentration-related increase; and (c) results in all evaluated test item concentrations should be within the range of the laboratory historical solvent control.

It is clastogenic and aneugenic if: (a) at least one test item concentration is statistically significantly increased compared with the concurrent solvent control; (b) a concentration-related increase occurs in at least one experiment trial; and (c) the results are outside the range of the laboratory historical solvent control data.

*Results*: Toxicity tests conducted using 10 concentrations showed phase separation occurred at the end of treatment in experimental trials: 4 h exposure time point ≥ 44.9 µg/mL (− S9) and ≥ 241 µg/mL (+ S9) (Table 7); 20 h exposure time point ≥ 250 µg/mL (− S9) (Table 8). No cytotoxicity was observed in the 4 h trials up to highest evaluated concentration which showed phase separation. In the 20 h experiment in the − S9, moderate cytotoxicity was observed at the highest evaluated concentration. No clear cytotoxic effects were able to be evaluated for cytogenetic damage.

**Table 7:** 2-Ethylhexyl acrylate. in vitro micronucleus test toxicity experiment - 4 hr exposure time

	Without S9		With S9	
Concentration (µg/mL)	CBPI per 500 cells	Cytostasis (%)	CBPI per 500 cells^a^	Cytostasis (%)
Solvent control	1.56	–	1.78	–
8.4	1.49	13.5	n.d.	n.d.
14.7	1.34	39.0	n.d.	n.d.
25.7	1.52	8.0	1.81	n.c.
44.9	***1.56***	***0.9***	1.74	5
78.6	***n.d.***	***n.d.***	1.74	5
138	***n.d.***	***n.d.***	1.75	4.6
241	***n.d.***	***n.d.***	***1.75***	***3.8***
421	***n.e***	***n.e***	***1.71***	***9.3***
737	***n.e***	***n.e***	***1.76***	***2.9***
1843	***n.e***	***n.e***	***1.66***	***15.6***

Bold italic indicates phase separation at the end of treatment by the unaided eye.

n.d. Not determined.

n.c. Not calculated as the CBPI was equal or higher than solvent control value.

a Mean value of two cultures.

**Table 8:** 2-Ethylhexyl acrylate. in vitro micronucleus test toxicity experiment - 20 hr exposure time.

	Without S9	
Concentration (µg/mL)	CBPI per 500 cells^a^	Cytostasis (%)
Acetone	1.63	–
4.4	n.d.	n.d.
7.6	1.71	n.c.
13.3	1.61	3.0
23.3	1.58	7.7
40.8	1.61	2.9
71.4	1.39	37.5
125	1.15	76.2
250	***n.e.***	***n.e.***
500	***n.e.***	***n.e.***

Bold italic indicates phase separation at the end of treatment by the unaided eye.

n.d. Not determined.

n.c. Not calculated as the CBPI was equal or higher than solvent control value.

n.e. Not evaluated due to strong cytotoxicity.

a Mean value of two cultures.

The micronuclei evaluation (Table 9) showed that in the absence of S9, two values at the 4 h time point were statistically significantly increased (8.4 and 44.9 µg/mL). Both values were clearly within the 95% control limit of the historical control data (0.06–1.19% micronucleated cells) and dose dependency tested via trend test was not statistically significant. Therefore, this finding was regarded as biologically irrelevant. At the 20 h time point in the absence of S9 after continuous treatment, and at the 4 h time point in the presence of S9 after pulse treatment, no relevant increases in the numbers of micronucleated cells were observed after treatment with 2-ethylhexyl acrylate.

**Table 9:** Summary of results of the 2-Ethylhexyl acrylate *in vitro* micronucleus test in human lymphocytes.

Preparation interval	Test item concentration in µg/mL	Proliferation index (CBPI)	Cytostasis (%*)	Micronucleated cells (%**)	95% Ctrl limit
**Exposure period 4 h without S9 mix**					
40 h	Solvent control^a^	1.56		0.40	0.06–1.19
	Positive control^b^	1.30	46.5	**18.85**^s^	3.92–25.34
	8.4	1.49	13.5	**1.10**^s^	
	14.7	1.34	39.0	0.60	
	25.7	1.52	8.0	0.75	
	44.9^PS^	1.56	0.9	**0.90**^s^	
**Exposure period 20 h without S9 mix**					
40 h	Solvent control^a^	1.63		0.40	0.00–1.11
	Positive control^c^	1.30	51.7	**2.20**^s^	1.47–5.89
	23.3	1.58	7.7	0.70	
	40.8	1.61	2.9	0.65	
	71.4	1.39	37.5	0.65	
**Exposure period 4 h with S9 mix**					
40 h	Solvent control^a^	1.77		0.90	0.08–1.38
	Positive control^d^	1.73	5.1	**6.65**^s^	0.70–10.20
	93.3	1.79	n.c.	0.25	
	163	1.85	n.c.	0.65	
	286^PS^	1.79	n.c.	0.80	

S The number of micronucleated cells is statistically significantly higher than corresponding control values.

PS Phase separation occurred at the end of treatment.

n.c. Not calculated as the CBPI was equal or higher than solvent control value.

* For the positive control groups and the test item treatment groups the values are related to the solvent controls

** The number of micronucleated cells was determined in a sample of 2000 binucleated cells

a Acetone 0.5% (v/v).

b MMC 1.0 µg/mL.

c Demecolcine 100 ng/mL.

d CPA 15.0 µg/mL.
